# Pneumolabyrinth nach Barotrauma bei Cochleaimplantat

**DOI:** 10.1007/s00106-025-01602-7

**Published:** 2025-04-08

**Authors:** S. Marti, N. Iob, D. Bächinger, C. Schlegel, A. Huber, J. Dlugaiczyk, A. Dalbert

**Affiliations:** 1 Klinik für Ohren‑, Nasen‑, Hals und Gesichtschirurgie, Universitätsspital Zürich, Rämistrasse 100, 8091 Zürich, Schweiz; 2 Universität Zürich, Zürich, Schweiz; 3 Klinik für Hals‑, Nasen‑, Ohren- und Gesichtschirurgie, Luzerner Kantonsspital, Luzern, Schweiz

**Keywords:** Sensorineurale Schwerhörigkeit, Schwindel, Rezidiv, Perilymphfistel, Eustachische Röhre, Hearing loss, sensorineural, Vertigo, Recurrence, Perilymphatic fistula, Eustachian tube

## Abstract

Bei einer 54-jährigen Patientin mit beidseitigen Cochleaimplantaten kam es links wiederholt zu einem Pneumolabyrinth nach Barotrauma. Dies resultierte jeweils in einer akuten peripher-vestibulären Unterfunktion und Cochleaimplantatdysfunktion. Eine als Ursache vermutete Perilymphfistel wurde bei 3 Ereignissen innerhalb von 6 Jahren jeweils operativ abgedeckt. Vorbeugend wurde schließlich eine Tubendilatation durchgeführt. Es handelt sich um die Erstbeschreibung eines durch Barotrauma induzierten, rezidivierenden Pneumolabyrinths nach Cochleaimplantation.

## Abstract (en)

We report the case of a 54-year-old female patient with bilateral cochlear implants suffering from recurrent left-sided pneumolabyrinth due to barotrauma. These episodes caused acute peripheral vestibular disorder and cochlear implant malfunction. Over a 6-year period, the suspected causative perilymphatic fistula was surgically sealed on three occasions. A preventative balloon dilation of the Eustachian tube was finally performed. This case report is the first to describe barotrauma-induced recurrent pneumolabyrinth in a patient with cochlear implants.

## Anamnese

Eine 54-jährige Patientin stellte sich 2017 und 2021 jeweils nach einer Flugreise notfallmäßig vor. Es bestand ein Zustand nach beidseitiger Cochleaimplantation – 2011 rechts und 2015 links – aufgrund einer schubweise verlaufenden, progredienten, an Taubheit grenzenden Innenohrschwerhörigkeit beidseits unklarer Ätiologie. In beiden Jahren trat während des Steigflugs akuter Drehschwindel auf, begleitet von linksseitigem Ohrdruck und einer Intoleranz gegenüber dem Sprachprozessor des linken Cochleaimplantats (CI) infolge von Geräuschverzerrungen. Bis auf einen hochfrequenten Tinnitus links und Nausea traten keine weiteren Symptome auf. Aus der Vorgeschichte sind rezidivierende Hörstürze beidseits bekannt, die bereits mit mehreren seriellen intratympanalen Dexamethason-Injektionen behandelt wurden (Seite unbekannt). Vor der zweiten Cochleaimplantation 2015 demarkierte sich im Video-Kopfimpulstest (vKIT) eine regelrechte Funktion beider lateraler Bogengänge (l-BG). Beidseits wurde die Elektrode „Standard“ mit einer Länge von 31,5 mm der Firma MED-EL Medical Electronics (Innsbruck, Österreich) implantiert. Bis auf die beidseitige Hörminderung war die Patientin gesund.

## Untersuchung

Die klinische Untersuchung gestaltete sich 2017 und 2021 wie folgt und, sofern nicht näher präzisiert, identisch:

Es zeigte sich ein inspektorisch reizloser Befund an der Implantationsstelle. Beidseits war die Ohrmikroskopie blande. Das Hennebert-Zeichen fiel negativ aus.

Im Jahr 2017 bestand ein Spontannystagmus Grad 2 nach rechts, während 2021 nur ein Kopfschüttelnystagmus nach rechts imponierte. Der Kopfimpulstest zeigte nach links jeweils Rückstellsakkaden; nach rechts fiel er normal aus. Eine vertikale Blickdeviation im Aufdecktest lag nicht vor. Im Romberg-Test ergab sich eine Falltendenz nach links, der Unterberger-Tretversuch war ohne Rotationsabweichung. Der weitere HNO-Status war blande.

## Diagnostik

**Abb. 1:**
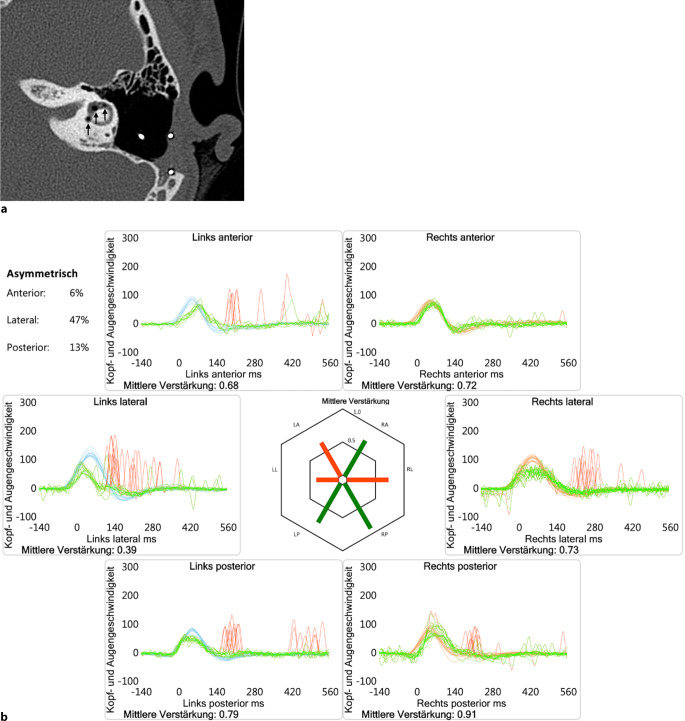
**a** Computertomographie Felsenbein links nativ von 06/2017; Axialschnitt auf Höhe des lateralen Bogengangs (l‑BG) links mit Lufteinschlüssen (*Pfeile*) und angeschnittener CI-Elektrode in der Mastoidektomiehöhle. **b** vKIT von 06/2017; bei der leichten „Gain-Reduktion“ des rechten l‑BG handelt es sich a.e. um ein „Push-Pull-Phänomen“ [1]

Sowohl eine Computertomographie 2017 (Schichtdicke 1 mm; Abb. 1a) als auch eine digitale Volumentomographie 2021 (Schichtdicke 0,15 mm) zeigten neben dem Nachweis von intralabyrinthären Lufteinschlüssen links eine korrekte Lage der CI-Elektroden beidseits.

Im vKIT (Abb. 1b) fiel 2017 eine neu aufgetretene peripher-vestibuläre Unterfunktion aller drei BG links auf („Gain“ < 1 plus Korrektursakkaden). Beim akuten Ereignis 2021 zeigte sich ebenfalls eine Unterfunktion aller drei BG links; zwischenzeitliche Verlaufskontrollen lagen leider nicht vor.

## Therapie und Verlauf

Es wurde 2017 und 2021 jeweils die Diagnose eines Pneumolabyrinths (PNL) bei vermuteter Perilymphfistel (PLF) links nach Barotrauma gestellt. In beiden Jahren erfolgte eine Tympanoskopie mit Abdeckung des runden und ovalen Fensters. Intraoperativ fanden sich jeweils eine korrekte Implantatlage und keine Hinweise auf eine PLF. Die vermutete PLF wurde 2017 am Luzerner Kantonsspital mit Bindegewebe und Fibrinkleber sowie 2021 am Universitätsspital Zürich mit Muskelgewebe und Fibrinkleber verschlossen. Die 2021 am Ende der Operation durchgeführten „Auditory-Nerve-Response-Telemetry-Messungen“ zeigten normale Impedanzen und Reizantworten auf allen Elektroden. Präoperative Messungen wurden nicht durchgeführt.

Das CI erlangte bei beiden Ereignissen (2017, 2021) nach dem Verschluss der PLF rasch die präoperativ vorhandene Funktion wieder, und die Patientin nahm die Geräuschverzerrungen nicht mehr wahr. Der „Switch-on“ erfolgte beim ersten Mal nach wenigen Tagen, beim zweiten Mal direkt postoperativ. Die Patientin berichtete jedoch über einen persistierenden Ohrdruck links und einen intermittierenden Drehschwindel mit Nausea bei schnellen Kopfbewegungen. Es wurde eine vestibuläre Physiotherapie etabliert, um die zentrale Kompensation zu fördern.

Anlässlich der Verlaufskontrolle (11/2021) drei Monate postoperativ erfolgte eine apparativ-vestibuläre Untersuchung, welche im vKIT eine regelrechte Funktion des anterioren BG (a-BG) links zeigte, während der l‑BG und posteriore BG (p-BG) weiterhin eine Unterfunktion mit vermindertem „Gain“ und Korrektursakkaden aufwiesen. Darüber hinaus wurde eine verminderte dynamische Sehschärfe bei Kopfdrehung nach links nachgewiesen. Der linke Sacculusreflex (zervikal vestibulär evozierte myogene Potenziale [cVEMP] bei 500 Hz Knochenleitung) war ausgefallen und der linke Utriculusreflex (okuläre VEMP bei 500 Hz Knochenleitung) im Seitenvergleich vermindert (Asymmetrie-Ratio 54 %).

Bei der Kontrolle zehn Monate postoperativ (07/2022) hatte sich nun auch die Funktion des p‑BG erholt, und es wurde lediglich noch eine Unterfunktion des l‑BG links (vKIT) dokumentiert. Die dynamische Sehschärfe war nach beiden Seiten normal als Zeichen einer zentralen Kompensation des gestörten vestibulookulären Reflexes links.

Im August 2023 kam es zum dritten Mal zu einem druckinduzierten PNL. Dieses Mal fuhr die Patientin mit der Zahnradbahn auf das Brienzer Rothorn (2350 m. ü. M., Höhendifferenz 1678 m, Fahrtdauer 60 min) und stellte sich erneut notfallmäßig mit vergleichbaren Symptomen wie 2017 und 2021 vor. Es erfolgte erneut eine endaurale Abdichtung des runden und ovalen Fensters am Universitätsspital Zürich, wobei diesmal Fasziengewebe und Fibrinkleber verwendet wurde.

Nach dem dritten Ereignis mit klarem zeitlichem Zusammenhang zu raschen Veränderungen des Luftdrucks wurde der Patientin die Durchführung einer Tubendilatation links empfohlen. Die Indikation basierte darauf, mittels einer möglichen Verbesserung der Mittelohrbelüftung die Entstehung eines weiteren PNL vorzubeugen.

Die Tubendilatation wurde mit dem Stryker-System (Fa. Stryker Corporation, Kalamazoo, Michigan, USA) durchgeführt, um eine Drucksteigerung im Mittelohr zu vermeiden. Hierbei konnte der Ballon während 2 min komplikationsfrei in der Tube belassen werden. Seit der Durchführung der Intervention (Follow-up von 17 Monaten) flog die Patientin zwar nicht mehr, aber sie konnte während ihres Skiurlaubs in Grimentz, Schweiz beschwerdelos mit der Gondel in die Höhe aufsteigen (1553 m. ü. M., Höhendifferenz 947 m, Fahrtdauer ca. 30 min).

## Diskussion

Bei einer PLF besteht eine Kommunikation zwischen dem Perilymphraum des Innenohrs und dem Mittelohr, dem Mastoid (äußere PLF) oder nach intrakraniell (innere PLF). Meistens tritt Perilymphe aus dem ovalen und/oder runden Fenster aus [6]. Laut Hidaka et al. entstehen lediglich etwa 6 % der traumatischen PLF durch Barotraumata. Die Mehrheit ist durch stumpfe (40 %) und penetrierende (35 %) Schädel-Hirn-Traumata verursacht [2]. Ein PNL entsteht typischerweise infolge einer PLF, über die Luft ins Innenohr eindringt. Solche Fälle wurden bereits bei Patienten mit CI beschrieben, etwa infolge von kräftigem Schnäuzen [5] oder der Nutzung einer CPAP-Maske [3]. Nach unserem Wissensstand wurde ein PNL, das durch eine rasche Luftdruckänderung (Barotrauma) während eines Höhenanstiegs verursacht wurde, bislang nicht beschrieben. Ebenso ist uns kein Fall mit rezidivierendem Verlauf bekannt.

Wir gehen davon aus, dass im dargestellten Fall ursächlich eine Tubenfunktionsstörung vorlag. Beim Abfall des Luftdrucks während eines Aufstiegs in höhere Lagen entsteht im Mittelohr, im Vergleich zum Epipharynx und zum äußeren Gehörgang, ein relativer Überdruck. Hierdurch öffnet sich die Eustachische Tube spontan, wodurch ein Druckausgleich erfolgen kann. Es erscheint wahrscheinlich, dass dies im vorliegenden Fall nicht funktionierte, sodass der Überdruck über eine PLF ins Innenohr abgeleitet wurde. Durch das resultierende PNL entstand schließlich eine akut peripher-vestibuläre sowie eine CI-Dysfunktion. Ein durch die CI-Elektrode bedingter „Locus minoris resistentiae“ begünstigte vermutlich die Entstehung einer PLF.

Trotz der Rezidive bleibt die Abdeckung der PLF aus unserer Sicht die sinnvollste Therapieoption. Zur Verbesserung der Tubenfunktion erfolgte schließlich zudem eine Tubendilatation. Studien belegen, dass diese Therapie eine wirksame und sichere Methode zur Behandlung einer Dysfunktion der Eustachischen Tube darstellt. Zu den Indikationen zählen unter anderem durch schnelle Luftdruckänderungen induzierte Symptome [4], einschließlich bei Personen mit CI, wie im beschriebenen Fall vorliegend.

## Fazit für die Praxis

- Dies ist die Erstbeschreibung eines rezidivierenden Pneumolabyrinths, das durch einen Höhenaufstieg bei einer Patientin mit Cochleaimplantat ausgelöst wurde.
- Bei akuter peripher-vestibulärer Störung und CI-Dysfunktion nach Druckänderungen sollte eine CT zur Abklärung eines PNL erfolgen.
- Das operative Abdecken der als Ursache vermuteten Perilymphfistel kann eine gute Prognose für die Wiederherstellung der CI- und vestibulären Funktion bieten.
- Eine vestibuläre Physiotherapie und Tubendilatation sind zu empfehlen.
